# Outcome of posterior decompression for spinal epidural lipomatosis

**DOI:** 10.1007/s00701-023-05814-0

**Published:** 2023-09-25

**Authors:** Michael Schmutzer-Sondergeld, Hanna Zimmermann, Raimund Trabold, Thomas Liebig, Christian Schichor, Sebastian Siller

**Affiliations:** 1 Department of Neurosurgery, LMU University Hospital, LMU Munich, Marchioninistrasse 15, 81377 Munich, Germany; 2 Department for Diagnostic and Interventional Neuroradiology, LMU University Hospital, LMU Munich, Marchioninistrasse 15, 81377 Munich, Germany

**Keywords:** Spinal epidural lipomatosis, Spinal stenosis, Posterior decompression

## Abstract

**Background:**

In contrast to osteoligamentous lumbar stenosis (LSS), outcome of surgical treatment for spinal epidural lipomatosis (SEL) is still not well defined. We present risk factors for SEL and clinical long-term outcome data after surgical treatment for patients with pure SEL and a mixed-type pathology with combined SEL and LSS (SEL+LSS) compared to patients with pure LSS.

**Methods:**

From our prospective institutional database, we identified all consecutive patients who were surgically treated for newly diagnosed SEL (*n* = 31) and SEL+LSS (*n* = 26) between 2018 and 2022. In addition, a matched control group of patients with pure LSS (*n* = 30) was compared. Microsurgical treatment aimed for posterior decompression of the spinal canal. Study endpoints were outcome data including clinical symptoms at presentation, MR-morphological analysis, evaluation of pain-free walking distance, pain perception by VAS-N/-R scales, and patient’s satisfaction by determination of the Odom score.

**Results:**

Patients with osteoligamentous SEL were significantly more likely to suffer from obesity (body mass index (BMI) of 30.2 ± 5.5 kg/m^2^, *p* = 0.03), lumbar pain (*p* = 0.006), and to have received long-term steroid therapy (*p* = 0.01) compared to patients with SEL+LSS and LSS. In all three groups, posterior decompression of the spinal canal resulted in significant improvement of these symptoms. Patients with SEL had a significant increase in pain-free walking distance during the postoperative course, at discharge, and last follow-up (FU) (*p* < 0.0001), similar to patients with SEL+LSS and pure LSS. In addition, patients with pure SEL and SEL+LSS had a significant reduction in pain perception, represented by smaller values of VAS-N and -R postoperatively and at FU, similar to patients with pure LSS. In uni- and multivariate analysis, domination of lumbar pain and steroid long-term therapy were significant characteristic risk factors for SEL.

**Conclusions:**

Surgical treatment of pure SEL and SEL+LSS allows significant improvement in pain-free walking distance and pain perception immediately postoperatively and in long-term FU, similar to patients with pure LSS.

## Introduction

Spinal epidural lipomatosis (SEL) is characterized by an excessive overgrowth of fat in the epidural space which can result in pronounced narrowing of the dural sac due to this space-occupying effect. SEL is responsible for up to 6% of all clinically relevant spinal stenoses [20]. The pathogenesis of epidural fat overgrowth remains essentially unclear [10, 19, 31]. On the one hand, SEL may be idiopathic in lean patients, but it may also be secondary to obesity and metabolic syndrome [13] or other endocrine diseases, such as Cushing’s disease [1, 7, 22]. Other risk factors include exogenous steroid intake and previous spinal surgery [30]. An association with neurosarcoidosis and inflammatory involvement of the factors TNFα and IL-1β has also been described [15]. Furthermore, studies have been able to show that depending on the etiology of SEL, different localizations in the spine are affected [10, 17, 18, 24]. However, the pathogenesis for this disease still appears mostly unclear.

Diagnosis is most accurately made by MRI imaging in T1-weighted sagittal and axial slices, and the extent of SEL is described by established grading scores according to Ishikawa et al. [14] and Borré et al. [8], respectively.

Therapeutic approaches address patient-specific symptoms and complaints. If a conservative therapy attempt based on physiotherapy, analgesia, and weight reduction does not improve symptoms or the patient develops new focal neurological deficits, surgical therapy should be considered. This usually consists of (unilateral) dorsal decompression using extended interlaminar fenestration with/without undercutting to the opposite side or hemilaminectomy.

However, several studies have shown inconsistent results after surgical therapy of SEL compared with pure LSS with regard to mobility and quality of life [6, 11, 29]. Therefore, in this retrospective study, we compared a larger collective of patients with pure SEL, pure lumbar spinal stenosis (LSS), and a mixed pathology (SEL+LSS) with SEL and LSS in different segments by means of postoperative outcome (walking distance), pain perception (VAS-N/VAS-R [27]), and surgical outcome (Odom criteria [23]).

## Materials and methods

### Patient population

After the approval of the institutional review board of the Ludwig-Maximilians-University in Munich (reference number 21-1084), the patient database of the Department of Neurosurgery was searched for all consecutive patients undergoing any surgical treatment of spinal epidural lipomatosis (SEL) or a mixed pathology with additional lumbar spinal stenosis (SEL+LSS) between 2018 and 2022. A matched control group of patients undergoing surgical treatment for pure lumbar spinal stenosis (LSS) during that retrospective period was identified. These patients were matched on the basis of comparable secondary diseases, age, and number of operated segments. Clinical and diagnostic evaluations were collected preoperatively and at routine follow-up evaluations (at dismission and last follow-up (FU)). Functional outcome analyses referred to pre- and post-operatively obtained data. All patients gave informed consent before surgical treatment.

### Magnetic resonance imaging

In all patients, preoperative magnetic resonance imaging (MRI) was available, our in-house standard protocol consisted of axial T2-weighted sequence (with slice thickness of 2 mm) and 3-dimensional T1-weighted sequences with axial, sagittal, and coronal reconstructions each (1.5- or 3.0-T scanners: Magnetom Symphony, Siemens, Erlangen; Signa HDxt; GE Healthcare, Little Chalfont, United Kingdom). Pure SEL and the SEL parts of patients with the mixed pathology were subdivided MR morphologically into grades I–III using the established classifications of Ishikawa et al. [14] and Borré et al. [8]. In both of these gradings, higher numbers correspond to a greater degree of epidural fat overgrowth. For the mixed pathology SEL+LSS, we included patients who had MR-morphologically demonstrable pure LSS in one spinal segment and pure SEL in one or more further spinal segment(s), and all pathologies were treated by posterior decompression. The stenosed segments in patients with SEL+LSS and pure LSS were MR morphologically graduated according to Schizas classification [26]. This classification describes the extent of LSS from A (little degree of stenosis), B (moderate stenosis), and C (severe stenosis) to D (extreme stenosis).

### Treatment protocol

In accordance with our in-house standards, patients with pure SEL, SEL+LSS, and pure LSS were managed by unilateral microsurgical fenestration with or without undercutting to the contralateral side or hemilaminectomy. The surgical procedure for SEL consisted of bony decompression by fenestration or hemilamiectomy and removal of the epidural fat so that the spinal canal and exiting nerve roots appeared free on all sides. In LSS, also bony decompression and removal of the hypertrophic ligamentum flavum were performed to relieve the spinal canal and nerve roots. Surgical methods were performed depending on the extent of SEL/LSS and surgeon’s risk assessment. No dorsal or ventral spondylodeses were performed.

### Outcome analyses

Pre-operative patients’ BMI was calculated with the following formula: bodyweight (in kg)/(body height (in m))^2^. Surgical results and follow-up analyses (at dismission and at FU) were assessed by quantitative instruments to determine pain sensation: evaluation of the visual numerical analog scale (VAS-N) at physical stress and at rest (VAS-R). Patients were asked about their pain level preoperatively and postoperatively (at discharge and at last FU). Furthermore, the Odom score [23] was used to determine patients’ satisfaction and symptom relief/persistence after surgery (at dismission and at last FU). Differences in Odom score between “discharge - FU” were calculated. Additionally, patients were asked about their pain-free walking distance (in meters) preoperatively, at dismission and last FU. Differences in walking distances at abovementioned time points were calculated and compared. Clinical long-term follow-up data (mean 47.8 ± 18.2 months, range: 9.6–63.6 months) of 82 patients were available. Four patients were lost to FU; one patient died during clinical FU.

### Risk assessment

Perioperative morbidity rates were determined according to all documented medical, neurological, and approach-related adverse events. Transient and permanent deficits were differentiated. Functional morbidity was analyzed separately.

### Statistical methods

Results were tested by using a 2-way analysis of variance (ANOVA), Student’s *t* and Fisher’s exact test. For risk factor analyses, uni- and multivariate tests were conducted. The corresponding control group with LSS-only was matched on the basis of the date of surgery, the number of spinal segments operated on, a comparable distribution of pathology in the lumbar spine, the age distribution of patients, and the physical characteristics. GraphPad PRISM8.0d software was used for statistical analysis (GraphPad, San Diego, CA, USA). Statistical significance was set *p* < 0.05.

## Results

### Patient characteristics, preoperative symptoms, and comorbidities

**Fig. 1:**
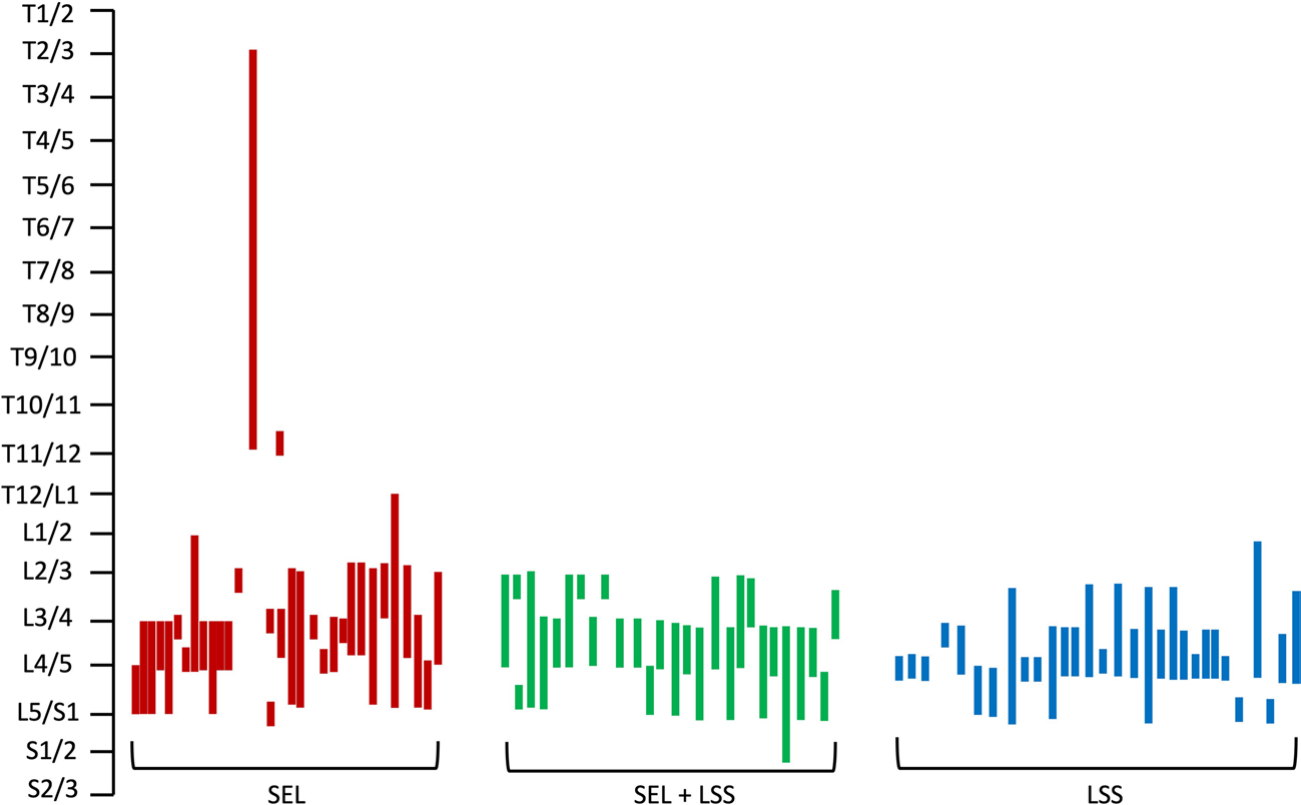
Distribution of segment numbers according to SEL, SEL+LSS, and LSS. T, thoracic spine; L, lumbar spine; S, sacral spine

**Fig. 2:**
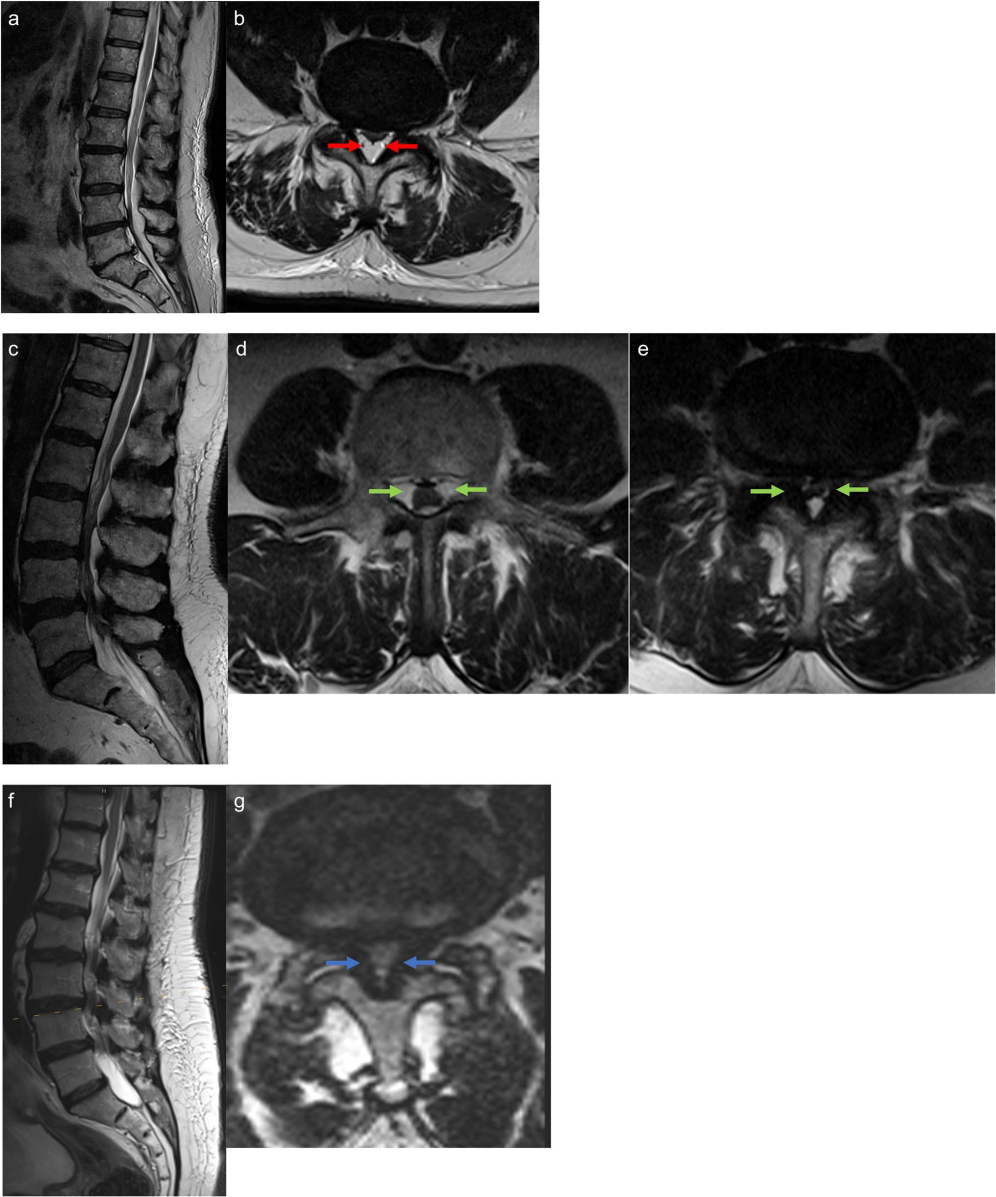
MR imaging (sagittal and axial) of patients with SEL (**a**, **b**), SEL+LSS (**c**, **d**, **e**), and pure LSS (**f**, **g**). SEL is characterized by abnormal overgrowth of adipose tissue in the epidural space (red arrows, **b**). The mixed pathology (SEL+LSS) shows in addition to excessive overgrowth of epidural fat in one segment (green arrows, **d**) also lumbar spinal stenosis due to hypertrophied ligamenta flava in another segment (green arrows, **e**), whereas in pure LSS, only ligamenta flava thickening is detectable (blue arrows, **g**)

Eighty-seven patients were included, of whom 27 were female (m:f = 2.2:1), and mean age was 69.8 ± 9.1 years (range: 37.6–83.3 years). Thirty-one of the patients (35.6%) had pure SEL, whereas 26 patients had a mixed type of SEL and LSS (29.9%). Additionally, a matched control group of 30 patients (34.5%) with pure LSS was analyzed. Baseline characteristics are displayed in Table 1 and did not significantly differ between the three cohorts. Furthermore, there was no difference in the mean number of operated spinal segments (*p* = 0.07). Figure 1 illustrates the distribution of operated spinal segments according to SEL, SEL+LSS, and LSS. Surgical procedure was standardized with all patients being surgically treated with a unilateral paravertebral-subperiostal approach for microsurgical fenestration or hemilaminectomy (on the clinically leading side) in combination with an undercutting procedure for bilateral decompression. Patients with pure SEL had a significantly (*p* = 0.03) increased BMI with 30.2 ± 5.5 kg/m^2^ and a significantly larger proportion of patients on long-term steroid therapy (*p* = 0.01). Although the rate of claudication symptoms was similar or higher in the SEL group compared to SEL+LSS and pure LSS, the preoperative pain-free walking distance was significantly longer in the SEL group. Furthermore, patients with SEL had more pre-operations compared to the other two patient groups (*p* = 0.05). More than 70% of these previous operations were performed in the same segment(s). The indications for preoperations in the index segments were LSS (87.5%) and lumbar disc herniation (12.5%). In the other segments, pure LSS had been previously operated. A similar distribution of previous operations was found for the other two patient groups: in the SEL+LSS group, the indications for preoperations in the index segment were LSS (75.0%) and lumbar disc herniation (25.0%). Previous surgeries in the pure LSS group were exclusively spinal stenosis. Median pre-operative symptoms duration was 6 months (range: 0.5–120 months): patient subgroups did not differ significantly (*p* = 0.6). Epidural lipomatosis in patients with pure SEL and SEL+LSS was classified using Borré and Ishikawa classifications. Here, both patient groups showed predominantly moderate to severe epidural fat overgrowth to both Borré and Ishikawa classification. Regarding the spinal canal stenosis classification according to Schizas, we found mainly severe and extreme stenoses for the SEL+LSS and pure LSS groups. For details, see Table 2. Figure 2 illustrates representative sagittal and axial MR imaging of patients with pure SEL, SEL+LSS, and LSS.

**Table 1:** Patients’ clinical and surgical parameters

*Parameter*	SEL	SEL+LSS	LSS	*p-value*
Clinical parameters				
Total, *n* (%)	31 (35.6)	26 (29.9)	30 (34.5)	
Sex, *n* (%)				
Male	22 (25.3)	21 (24.1)	17 (19.5)	0.6
Female	9 (10.3)	5 (5.7)	13 (14.9)	0.07
Age (yrs)	69.2 ± 7.1	72.0 ± 8.9	68.5 ± 10.8	0.3
Body mass index (kg/m^2^)	30.2 ± 5.5	27.5 ± 3.8	27.1 ± 4.8	**0.03**
Steroid long-term medication, *n* (%)	7 (8.0)	2 (2.3)	0	**0.01**
Number of segments	2.8 ± 1.8	2.4 ± 0.8	2.0 ± 1.1	0.07
Walking distance preoperatively (m)	400.3 ± 636.7	161.3 ± 154.5	163.3 ± 127.3	**0.04**
VAS-N preoperatively	6.2 ± 0.7	6.4 ± 0.8	6.2 ± 1.2	0.4
VAS-R preoperatively	5.9 ± 0.6	6.2 ± 0.9	5.8 ± 1.4	0.3
Duration of stay (d)	7.4 ± 3.0	8.3 ± 3.1	7.5 ± 2.1	0.4
Symptoms duration (mo)	15.1 ± 23.4	18.2 ± 20.3	13.0 ± 19.0	0.6
Follow-up (mo)	30.5 ± 16.7	48.7 ± 14.2	48.6 ± 16.8	**0.0002**
Surgical parameters				
Surgery side, *n* (%)				
Right	13 (14.9)	12 (13.8)	18 (20.7)	0.3
Left	17 (19.5)	12 (13.8)	12 (13.8)	0.5
Both-sided	1 (1.1)	2 (2.3)	0	0.3
Previous surgery, *n* (%)	11 (13.8)	5 (9.2)	3 (3.4)	0.05
Index segment(s)	8 (72.7)	4 (80.0)	2 (66.7)	0.9
Other segment(s)	3 (27.3)	1 (20.0)	1 (33.3)	
Re-surgery, *n* (%)	2 (2.3)	0	1 (1.1)	0.4
Period surgery-discharge (d)	5.8 ± 2.2	6.3 ± 2.5	5.9 ± 2.1	0.7
Surgical time (min)	177.6 ± 64.2	172.3 ± 70.8	168.9 ± 58.0	0.9
Bloodloss (ml)	454.7 ± 513.2	372.3 ± 403.2	339.3 ± 335.4	0.5

*n* number of patients, *m* meter, *VAS-N* visual numerical analog scale at physical stress, *VAS-R* visual numerical analog scale at rest (VAS-R), *d* days, *mo* months, *min* minutes, *ml* milliliter, *yrs* years

bold values represent statistical significant values as written in "Methods" (*p*<0.05)

**Table 2:** Graduation of patients with pure SEL and SEL parts in mixed pathology according to Borré and Ishikawa classification. LSS parts of the mixed group and pure LSS were specified according to Schizas classification

	SEL *n* = 31 (%)	SEL+LSS *n* = 26 (%)	LSS *n* = 30 (%)	*p-value*
Borré classification [8], *n* (%)				
Normal 0	0	0		0.99
SEL I	0	1 (3.8)		0.4
SEL II	29 (93.5)	18 (69.2)		0.07
SEL	2 (6.5)	7 (26.9)		0.06
Ishikawa classification [14], *n* (%)				
Sagittal				
Grade 1	4 (12.9)	2 (7.7)		0.7
Grade 2	17 (54.8)	19 (73.1)		0.2
Grade 3	10 (32.3)	5 (16.1)		0.4
Axial				
Grade 1	2 (6.5)	3 (11.5)		0.6
Grade 2	18 (58.1)	16 (61.5)		0.99
Grade 3	11 (35.4)	7 (26.9)		0.6
Schizas classification[26], *n* (%)				
A1		0	0	0.99
A2		0	0	0.99
A3		0	0	0.99
A4		0	0	0.99
B		0	3 (10.0)	0.2
C		20 (76.9)	13 (43.3)	**0.01**
D		6 (23.1)	14 (46.7)	0.1

bold values represent statistical significant values as written in "Methods" (*p*<0.05)

Patients with pure SEL more often presented with lumbar pain (*p* = 0.006), gait disturbance (*p* = 0.002), and claudication (*p* = 0.0001) compared to patients with SEL+LSS and pure LSS. Other complaints such as paresis, sensory disturbances, or radicular pain were equally distributed amongst all 3 patient groups. Both symptoms specific to SEL and the equally distributed complaints improved after surgical treatment already at the time of discharge and in the further course, so that only a few non-specific symptoms were still present in patients at last FU. For details, see Table 3.

**Table 3:** Distribution of symptoms in patients with SEL, SEL+LSS, and LSS preoperatively, at dismission and at last FU

*Parameter*	SEL *n* = 31 (%)	SEL+LSS *n* = 26 (%)	LSS *n* = 30 (%)	*p-value*
Symptoms preoperatively				
Paresis	18 (20.7)	9 (10.3)	16 (18.4)	0.2
Sensory disorder	22 (25.3)	16 (18.4)	14 (16.1)	0.1
Lumbar pain	28 (32.2)	18 (20.7)	16 (18.4)	**0.006**
Radicular pain	29 (33.3)	23 (26.4)	26 (29.9)	0.7
Bladder/sphincter disorder	1 (1.1)	2 (2.3)	0	0.3
Gait disturbance	9 (10.3)	2 (2.3)	0	**0.002**
Spasticity	0	0	0	0.99
Claudication	30 (34.5)	26 (29.9)	20 (23.0)	**0.0001**
Symptoms at dismission				
Paresis	9 (10.3)	5 (5.7)	5 (5.7)	0.5
Sensory disorder	7 (8.0)	4 (4.6)	5 (5.7)	0.7
Lumbar pain	2 (2.3)	2 (2.3)	1 (1.1)	0.8
Radicular pain	4 (4.6)	2 (2.3)	0	0.1
Bladder/sphincter disorder	1 (1.1)	0	0	0.4
Gait disturbance	2 (2.3)	1 (1.1)	0	0.4
Spasticity	0	0	0	0.99
Claudication	2 (2.3)	1 (1.1)	1 (1.1)	0.8
Symptoms at last FU				
Paresis	6 (6.9)	3 (3.4)	3 (3.4)	0.6
Sensory disorder	6 (6.9)	4 (4.6)	5 (5.7)	0.9
Lumbar pain	4 (4.6)	3 (3.4)	1 (1.1)	0.4
Radicular pain	1 (1.1)	2 (2.3)	0	0.2
Bladder/sphincter disorder	1 (1.1)	0	0	0.4
Gait disturbance	0	0	0	0.99
Spasticity	0	0	0	0.99
Claudication	0	0	1 (1.1)	0.4

bold values represent statistical significant values as written in "Methods" (*p*<0.05)

Table 4 lists main co-morbidities of patients with SEL, SEL+LSS, and LSS. Cardiovascular diseases, such as arterial hypertension, coronary artery disease (CAD)/peripheral artery disease (PAD), and associated risk factors (diabetes mellitus, nicotine abuse), were predominant in all 3 groups.

**Table 4:** Distribution of comorbidities of patients with SEL, SEL+LSS, and LSS

Parameter	SEL *n* = 31 (%)	SEL+LSS *n* = 26 (%)	LSS *n* = 30 (%)	*p*-value
No co-morbidity	1 (1.1)	2 (2.3)	3 (3.4)	0.6
Diabetes mellitus	8 (9.2)	6 (6.9)	7 (8.0)	0.9
Arterial hypertension	26 (29.9)	16 (18.4)	18 (20.7)	0.08
Nicotine abuse	10 (11.5)	5 (5.7	5 (5.7)	0.3
Peripheral artery disease	9 (10.3)	2 (2.3)	5 (5.7)	0.1
Coronary artery disease	15 (17.2)	8 (9.2)	16 (18.4)	0.2
Carpal tunnel syndrome	1 (1.1)	0	0	0.4
Osteoarthritis knee	6 (6.9)	2 (2.3)	5 (5.7)	0.4
Osteoarthritis hip	2 (2.3)	2 (2.3)	3 (3.4)	0.9
Lumbar spine syndrome	7 (8.0)	5 (5.7)	10	0.4
Osteoporosis	0	0	0	0.99
Rheumatoid arthritis	0	1 (1.1)	0	0.4
Obesity	13 (14.9)	8 (9.2)	9 (10.3)	0.5
Alcohol abuse	3 (3.4)	2 (2.3)	2 (2.3)	0.9
Cancer disease	1 (1.1)	4 (4.6)	4 (4.6)	0.3
Spine segmentation disorder	0	0	0	0.99
Tethered cord	0	0	0	0.99
Myelomeningocele	0	0	0	0.99
Chiari malformation	0	0	0	0.99
Epileptic seizures	0	0	2 (2.3)	0.1
Stroke	0	2 (2.3)	2 (2.3)	0.3
Clubfoot	0	0	0	0.99
Heart malformation	0	1 (1.1)	0	0.4
Hydrocephalus	0	0	0	0.99
Secondary spinal tumor	0	0	0	0.99

## Outcome

### Walking distance difference

**Fig. 3:**
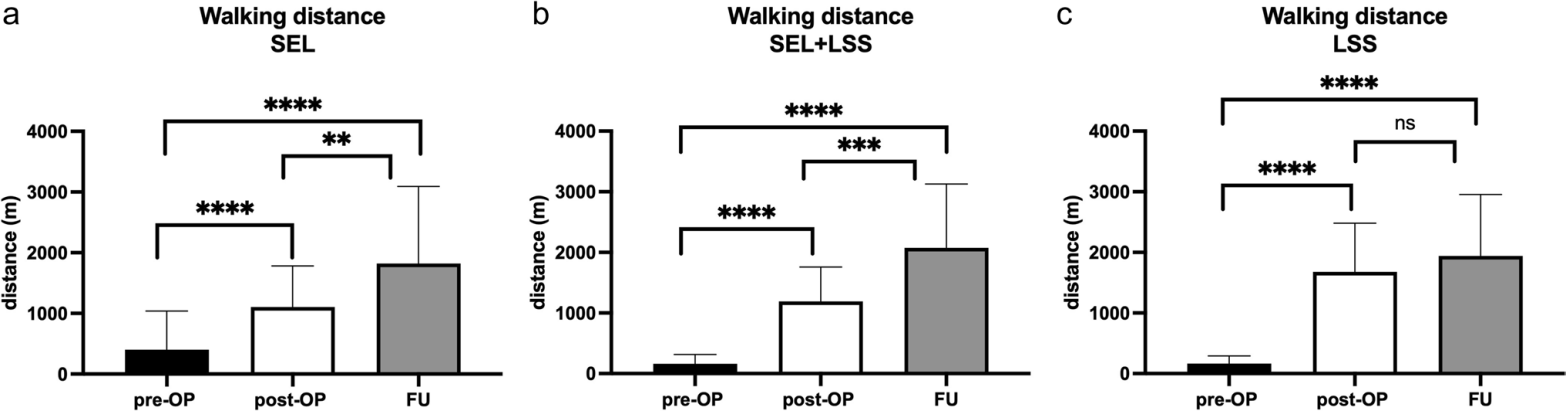
Illustration of walking distance differences for patients with pure SEL (**a**), SEL+LSS (**b**), and pure LSS (**c**). ^****^p < 0.0001, ^***^p = 0.001, ^**^p = 0.008

To evaluate the surgical success, patients were asked about their walking distance both preoperatively (see Table 1) and postoperatively at discharge and at FU. Preoperative pain-free walking distance of patients with pure SEL was significantly longer (*p* = 0.04) with 400.3 ± 636.7 m compared to patients with SEL+LSS (161.3 ± 154.5 m) and patients with pure LSS (163.3 ± 127.3 m). Patients with pure SEL (Fig. 3a) reported a significant improvement of the pain-free walking distance to 1106.0 ± 674.3 m at discharge (*p* < 0.0001) and further to 1821 ± 1272 m at FU (*p* < 0.0001). Patients with SEL+LSS also reported a significant improvement of the walking distance to 1192.0 ± 567.2 m at discharge (*p* < 0.0001) and to 2075 ± 1055 m at FU (*p* < 0.0001). For both patients in the SEL and SEL+LSS groups, there was also further significant improvement in pain-free walking distance between FU and discharge (*p* = 0.008 resp. *p* = 0.001). Patients with pure LSS also had a significant improvement in walking distance to 1677.0 ± 804.1 m at discharge (*p* < 0.0001). At the FU time point, the mean pain-free walking distance was 1940.0 ± 1014.0 m. At discharge, patients with pure LSS had a longer walking distance compared to patients with pure SEL (*p* = 0.005) and patients with SEL+LSS (*p* = 0.02). Walking distances at FU time point did not differ between patient groups.

### Evaluation of VAS-N/-R

**Fig. 4:**
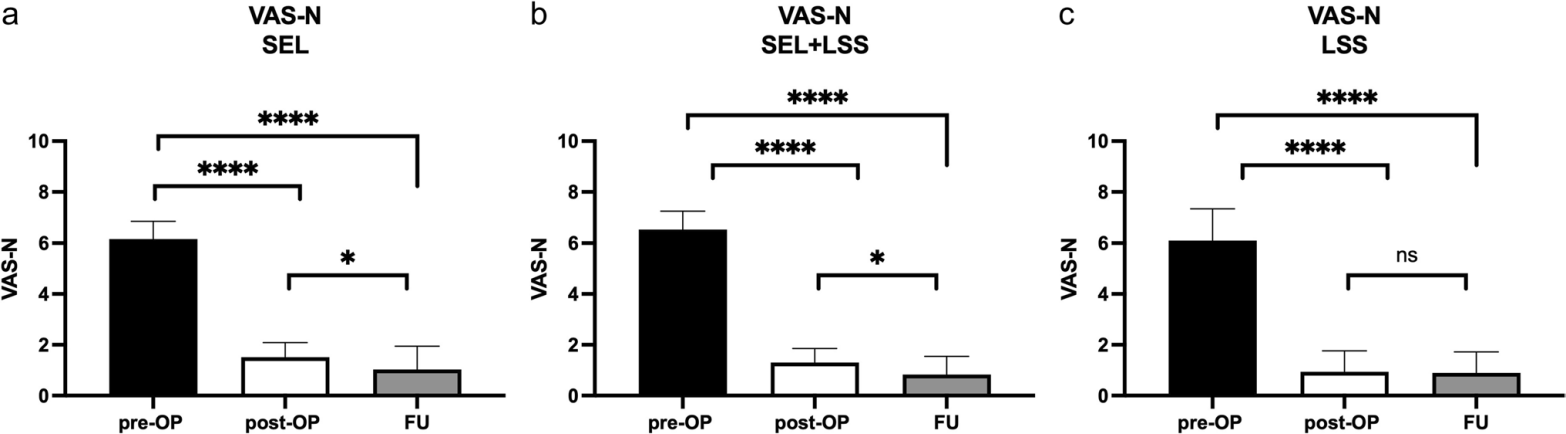
VAS-N for patients with SEL (**a**), SEL+LSS (**b**), and LSS (**c**) preoperatively, postoperatively, and at FU. ^****^p < 0.0001, ^*^p = 0.01

**Fig. 5:**
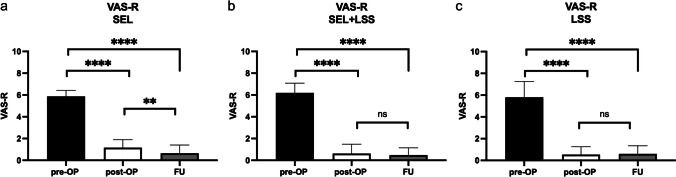
VAS-R for patients with SEL (**a**), SEL+LSS (**b**), and LSS (**c**) preoperatively, postoperatively, and at FU. ^****^p < 0.0001, ^**^p = 0.008

Another outcome parameter was the evaluation of the visual numerical analog scale (VAS-N) at physical stress and at rest (VAS-R). Patients were asked about their pain preoperatively (Table 1) and postoperatively (at discharge and at FU). The comparisons for VAS-N/-R for patients with pure SEL (Figs. 4a and 5a), SEL+LSS (Figs. 4b and 5b), and pure LSS (Figs. 4c and 5c) are shown. Pain reduction was reflected in both reduced VAS-N and VAS-R scores. The preoperative VAS-N/-R did not differ distinctly between the 3 patient groups at a median of 6 each (*p* = 0.3 resp. *p* = 0.4). We found a significant reduction in VAS-N at the postoperative (median: 1) and FU (median: 1) time points compared with the preoperative condition in all 3 patient groups (*p* < 0.0001). Furthermore, patients with pure SEL and SEL+LSS showed a significant reduction in VAS-N between postoperative and FU survey (*p* = 0.01). This comparison was insignificant in patients with pure LSS (*p* = 0.8). A comparison of the VAS-R also showed a significant reduction at the postoperative and FU time points for all 3 patient groups (*p* < 0.0001) to a median of 1 and 0. Similarly, patients with pure SEL also showed a significant difference with a renewed reduction between postoperative and FU assessment (*p* = 0.008), whereas patients with SEL+LSS and pure LSS had similar VAS-R values between postoperative and FU time points (*p* = 0.5 resp. *p* = 0.7).

### Evaluation of the Odom score

**Fig. 6:**
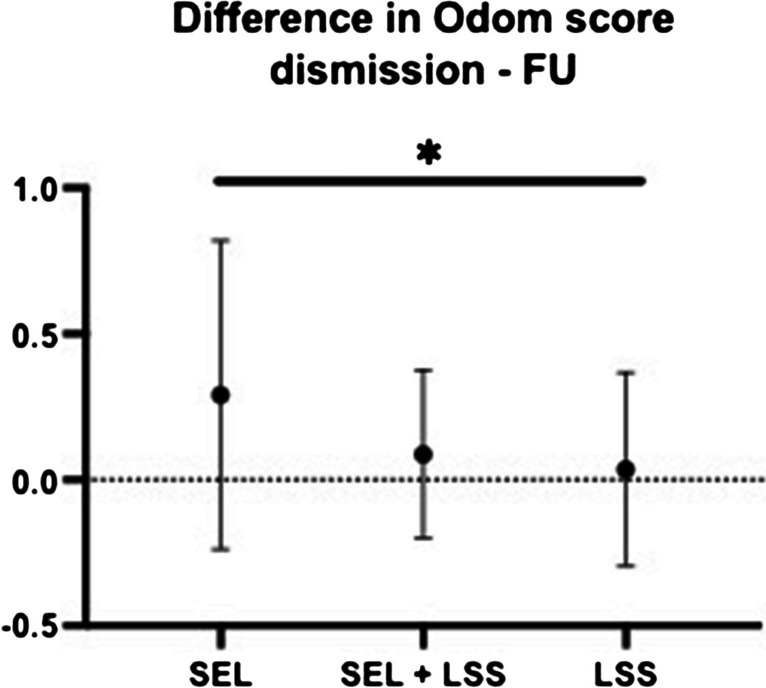
Odom score differences (*p = 0.04)

Using the Odom criterion, the success of surgical therapy within the 3 patient groups was analyzed postoperatively at discharge and at FU (for details, see Table 5). The majority of patients showed a satisfying surgical outcome (excellent and good) at the time of discharge without significant differences between the patient groups. However, there was a tendency for the SEL-only group to have a more frequent “good” surgical outcome than patients with mixed pathology and pure LSS (*p* = 0.01). At the FU time point, there was a predominantly “excellent” surgical outcome in all 3 groups. The difference between Odom scores at discharge and FU was significantly greater for patients with pure SEL compared to the other two patient groups (*p* = 0.04, Fig. 6).

**Table 5:** Distribution of Odom scores for patients with SEL, SEL+LSS, and LSS at dismission and FU

*Odom score at dismission*				
*Parameter*	SEL *n* = 31 (%)	SEL+LSS *n* = 26 (%)	LSS *n* = 30 (%)	*p-value*
1) Excellent	12 (13.8)	20 (23.0)	20 (23.0)	0.3
2) Good	17 (19.5)	5 (5.7)	9 (10.3)	0.01
3) Fair	2 (2.3)	1 (1.1)	1 (1.1)	0.8
4) Poor	0	0	0	0.99
*Odom score at FU*				
*Parameter*	SEL *n* = 31 (%)	SEL+LSS *n* = 23 (%)	LSS *n* = 28 (%)	*p-value*
1) Excellent	20 (24.4)	19 (23.2)	20 (24.4)	0.3
2) Good	10 (12.2)	4 (4.9)	8 (9.8)	0.5
3) Fair	1 (1.2)	0	0	0.4
4) Poor	0	0	0	0.99

### Surgical morbidity and revision surgeries

The intraoperative course was free of complications in 80 cases (92.0%). In 7 cases (8.0%), an iatrogenic dura opening occurred, which could be closed again sufficiently and tightly by suturing and by the attachment of dural seal devices.

In a total of 87 surgical interventions, 3 revision surgeries (3.4%) were performed: 2 patients with SEL and one patient with LSS. Two of these revision surgeries (one fenestration and one fenestration with undercutting, one patient in SEL and one patient in LSS group) were due to insufficient decompression of the spinal canal with a persistent residual stenosis, which was diagnosed by new MRI due to persistent radicular pain and slight paresis post-operatively with failure to improve symptoms despite dexamethasone and adequate pain medication. These MRI scans were performed after a mean period of 18.45 days. One patient underwent revision (hemilaminectomy) in the SEL group after 0.6 days due to CSF leakage, which appeared to be a new leakage after initially intra-operatively sufficiently closed CSF leakage by suture and closure materials. Revision surgeries were performed after a median period of 3.9 days (range: 0.6–33.0 days). Four patients were lost to FU and one patient died during FU due to urosepsis, which was not related to spinal surgery.

### Factors associated with SEL

To determine factors associated with a higher likelihood for the presence of a SEL, certain parameters were compared in univariate logistic regression. Local lumbar pain (*p* = 0.007), gait disturbance (*p* = 0.0007), long-term steroid therapy (*p* = 0.006), and arterial hypertension (*p* = 0.03) were found to be significantly associated factors. In multivariate analysis, lumbar pain (*p* = 0.008), gait unsteadiness (*p* = 0.001), and steroid medication (*p* = 0.001) were further confirmed as significantly associated factors. For details, see Table 6.

**Table 6:** Uni- (A) and multivariate (B) analysis for risk factors and characteristics for patients with SEL

Variable	Odds ratio	95% CI	*p*-value
(A) Univariate analysis			
Sex (male vs female)	1.1	0.4508 to 3.107	0.8
Symptoms preoperatively			
Paresis	1.7	0.7116 to 4.233	0.2
Sensory disorder	2.1	0.8480 to 5.602	0.1
Lumbar pain	6.0	1.849 to 27.39	**0.007**
Radicular pain	2.1	0.4632 to 14.53	0.4
Bladder/sphincter disorder	0.9	0.04086 to 9.770	0.9
Gait disturbance	11.1	2.594 to 76.38	**0.0007**
Claudication	6.5	1.159 to 122.8	0.08
Co-morbidities			
Steroid long-term therapy	7.9	0.5614 to 4.017	**0.006**
Diabetes mellitus	1.1	0.4037 to 3.145	0.8
Arterial hypertension	3.4	1.193 to 11.12	**0.03**
Nicotine abuse	2.2	0.7877 to 6.141	0.1
Peripheral artery disease	2.9	0.9500 to 8.981	0.06
Coronary artery disease	1.2	0.5159 to 3.032	0.6
Osteoarthritis knee	1.7	0.4935 to 5.589	0.4
Osteoarthritis hip	0.7	0.09652 to 3.494	0.7
Lumbar spine syndrome	0.8	0.2716 to 2.179	0.7
Obesity	1.7	0.6613 to 4.149	0.3
Alcohol abuse	1.4	0.2594 to 6.748	0.7
Cancer disease	0.2	0.01051 to 1.170	0.1
(B) Multivariate analysis			
Lumbar pain	2.7	0.07219 to 0.4630	**0.008**
Gait disturbance	3.4	0.1863 to 0.7170	**0.001**
Steroid long-term therapy	3.3	0.1903 to 0.7619	**0.001**
Arterial hypertension	1.7	-0.02849 to 0.3513	0.09

bold values represent statistical significant values as written in "Methods" (*p*<0.05)

## Discussion

This study is one of the largest retrospectively collected investigations on the systematic analysis of surgical success in spinal epidural lipomatosis. For this purpose, 31 patients with pure lumbar SEL and 26 patients with both MRI-proven SEL and lumbar spinal stenosis (LSS) in another segment were analyzed. As a control group, 30 patients with pure LSS and similar segmental extent of the stenosis as well as clinical parameters were included. An important new finding was not only the comparison of pure SEL with LSS, but also with a third group of patients (SEL+LSS) who had both lumbar spinal stenosis and epidural lipomatosis in another segment. We believe it is important to include and describe this third group with a mixed pathology because this is a rather frequent finding in the daily clinical routine of a high-volume spine center, and it still remains controversial whether or not to include the SEL level in the decompression procedure or not. We reconfirmed that patients with SEL had significantly increased BMI compared to patients with LSS alone and the combined pathology (SEL+LSS) (*p* = 0.03). Furthermore, patients with SEL reported significantly more localized lumbar pain (*p* = 0.006) and had long-term steroid therapy than patients in the other two groups (*p* = 0.01). Furthermore, we demonstrated that patients with SEL, but also patients with SEL+LSS experienced a significant reduction in pain-free walking distance in the postoperative course (*p* < 0.0001) and also a significant pain relief represented by VAS-N/-R—similar to pure LSS patients. We therefore assume that decompression of LSS and SEL levels in patients with a mixed pathology can lead to good postoperative outcomes.

### Clinical characteristics

The pathogenesis of epidural fat overgrowth remains largely unclear. Metabolic alteration in favor of increased abnormal fat metabolism represents an important risk factor [10, 19, 31]. Similarly, excessive visceral adiposity represents another risk factor [16, 21]. Studies have shown that different localizations of SEL may result from endogenous excessive steroid production and exogenous steroid intake [17, 18, 24]. Patients of our study population with pure SEL compared to patients with SEL+LSS and pure LSS had a significantly increased BMI as already reported [3, 9, 17, 18]. Furthermore, we found that patients with pure SEL were significantly more likely to have steroids in their permanent medication. This has also been shown previously in other studies [10, 11]. In our study, despite the significantly increased BMI of 30.2 ± 5.5kg/m^2^ in SEL patients, no association between obesity and the occurrence of SEL could be shown in univariate analysis. Similar results have also been published previously [2, 4].

Patients with pure SEL presented significantly more often local lumbar back pain (*p* = 0.006) and claudication (*p* = 0.0001) prior to surgery when compared to patients with mixed pathology (SEL+LSS) and pure LSS, although the extent of claudication severity was lower in the pure SEL group (median pain-free walking distance 150 m vs 100 m and 100 m; *p* = 0.04). The higher rate of gait disturbance (*p* = 0.002) in the pure SEL group is mainly provoked by the higher rate of thoracic location of pathology. Altogether, the presenting symptomatology in the patients with pure SEL in our cohort is typical for a compressive pathology, and we therefore conclude that pathological epidural fat overgrowth (like SEL) can cause symptoms of spinal stenosis even in the absence of other compressive pathologies like thickening of the ligamenta flava or boney narrowing of the canal. This is in line with other studies [5, 25, 28] describing local effects damaging the spinal cord or nerve roots, such as radiculopathy or myelopathy. In our collective, patients of the 3 groups had a similar symptom duration until surgical treatment of mean 15.4 ± 20.9 months (*p* = 0.6). A likewise non-acute development of these symptoms has been described previously [12, 32].

Our study is the first to also describe the group of patients with a mixed-type pathology with a combination of SEL and LSS in different spinal levels. With identification of 26 patients with SEL+LSS during our 6-year observational period, the mixed type pathology has a similar incidence as the type with pure SEL (31 patients). Patients in the SEL+LSS group had lower BMI than patients with SEL only. Main preoperative symptoms were—similar to those in patients with pure SEL—radicular pain as well as claudication and only to a lesser extent local lumbar back pain and gait unsteadiness. However, compared with pure LSS patients, back pain, claudication, and gait instability were more frequent in the mixed pathology group. The reduced pain-free walking distance for the SEL+LSS group was similar to that for pure LSS and thus significantly lower than for SEL. These symptoms all improved uniformly postoperatively and in further FU. Because our study was the first to include a group with both pathologies in different segments, a direct comparison with the existing literature is difficult here. However, since similar complaints as in our SEL and LSS groups were described in patients with pure SEL [6, 10] and pure LSS [6], similarity and reproducibility can definitely be assumed in these cases, so that the mixed pathology group can be considered a separate entity. It should also be mentioned that SEL+LSS is a rather frequent finding in everyday life compared to pure SEL. It is still controversial, whether to include the lipomatosis level or not in the surgical procedure [6, 10, 11]. We included this group and always the SEL levels as well in decompression, which led to a good outcome. Therefore, we reasoned that SEL+LSS with its resulting limitations and symptoms is relevant in patients’ lives and we would also recommend decompression in SEL levels.

### Surgical therapy and outcome

Surgical treatment of SEL usually consists of microsurgical posterior decompression of the spinal canal and removal of epidural fat via unilateral or bilateral fenestration or hemilaminectomy in the absence of instability. These surgical approaches have been used in all our patients with both pure SEL, SEL+LSS, and pure LSS. The symptoms of back pain, gait unsteadiness, and claudication, which were dominant in pure SEL, improved with surgical therapy, so that these symptoms were present only to a minor extent at the postoperative and FU time points. Improvement of symptoms in patients with pure SEL was similar to that of patients with pure LSS, underlining the value of surgical treatment for a spinal stenosis caused by pure pathological overgrowth of fat in the epidural space. The patients with SEL+LSS also showed a significant improvement of these symptoms, and we therefore believe that it is important in this group of patients to surgically address both the levels of LSS and SEL. The symptoms of back pain, gait unsteadiness, and claudication symptomatology, which were dominant in pure SEL, improved with surgical therapy, so that these symptomatologies were present only to a minor extent at the postoperative and FU time points. The patients with SEL+LSS and pure LSS also showed a significant improvement of these symptoms. Bayerl et al. [6] were also able to show a clear and, above all, comparably good improvement in the symptoms of SEL and LSS.

Furthermore, we showed that patients with pure SEL tended to have had prior surgery for spinal stenosis or disc herniation more frequently in the index segment, suggesting that previous operations may be a risk factor for developing SEL. However, this can only be assumed due to the small patient cohort.

In our study, we demonstrated a significant reduction in VAS-N and -R in patients with pure SEL at the postoperative and FU time points. This was also reflected in the significant increase in pain-free walking distance. Patients with SEL+LSS also had a significant reduction in VAS-N at the FU time point. Patients with pure LSS also showed a significant reduction of VAS-N and -R at the FU time point compared to the preoperative status, but without significant further improvement after surgical treatment. Bayerl et al. [6] showed a comparable significant improvement for both patients with SEL and pure stenosis compared to preoperative findings.

The Odom score survey showed postoperatively and at the FU time point mainly patients with “excellent” and “good” outcome in all 3 patient groups of our cohort. In the comparison between postoperative and FU time point, the difference for patients with pure SEL was significantly larger compared to the other two groups. Bayerl et al. [6] also demonstrated a similar postoperative distribution of the Odom score for both patients with pure SEL and pure stenosis. In contrast to these results, other studies showed a worse outcome after SEL surgery in terms of pain-free walking distance and quality of life [11, 29].

## Revisions

The extent of complications requiring surgery in our study was comparably low with 3 out of 87 operations (3.4%), as already described in other studies [6, 31]. Intraoperative iatrogenic dura openings could be closed sufficiently; however, one case needed an early revision surgery due to new CSF leakage postoperatively.

## Limitations

Due to the retrospective study design, only data already collected at the pre- and postoperative as well as FU time points could be evaluated, and a possible selection bias cannot be excluded. Additionally, the relative small sample size of 87 patients in total and only about 30 patients per group holds the potential to overinterpret the results. Furthermore, no quality of life questionnaires were available, which might have allowed additive statements on the outcome after surgery in SEL and mixed pathology. In this respect, further prospective and even larger multicenter studies may allow an extensive evaluation of the surgical outcome and risk assessment for SEL.

## Conclusion

To our knowledge, this is one of the largest retrospective studies evaluating surgical outcome in patients with pure SEL and SEL with concomitant presence of LSS in another segment. We could show that posterior decompression in SEL and mixed pathologies has a significant effect on improvement of pain-free walking distance, pain perception, and patient satisfaction after surgery in the short- and long-term FU. This study confirms the success of surgery for SEL with significant improvement of preoperative symptoms, confirming surgery to be an effective treatment tool in symptomatic SEL.

## Data Availability

Informed consent was obtained from all individual participants included in the study.
